# Supplementary material for: Detection of diabetic macular oedema: validation of optical coherence tomography using both foveal thickness and intraretinal fluid
Source: PeerJ. 2015 Nov 10;3:e1394. doi: 10.7717/peerj.1394 (PMC4647548; doi:10.7717/peerj.1394)
Supplement: Supplemental Information 1 — FT, foveal thickness; IRF, intraretinal fluid. [file peerj-03-1394-s001.docx]

SUPPLEMENTAL MATERIAL:

Predictive model:

$$\frac{e^{FT\cdot0.016+\left( 1-IRL \right)\cdot\left( -3.576 \right)-3.749}}{1+e^{FT\cdot0.016+(1-IRL)\cdot\left( -3.576 \right)-3.749}}$$

FT, foveal thickness; IRF, intraretinal fluid.
